# MetaMask for thermal–humidity regulation in cold-environment facial protection

**DOI:** 10.1093/nsr/nwaf573

**Published:** 2025-12-15

**Authors:** Qifei Wang, Yufeng Yang, Qian Liang, Jihong Yu

**Affiliations:** State Key Laboratory of Inorganic Synthesis and Preparative Chemistry, College of Chemistry, Jilin University, China; International Center of Future Science, Jilin University, China; School of Fine Arts, Central China Normal University, China; School of Fine Arts, Central China Normal University, China; State Key Laboratory of Inorganic Synthesis and Preparative Chemistry, College of Chemistry, Jilin University, China; International Center of Future Science, Jilin University, China

Although cold conditions are often overshadowed by global warming, the unprecedented Northern Hemisphere cold waves, driven by the ‘Warm Arctic, Cold Continents’ pattern may pose a significant yet underestimated threat to global public health [1]. In cold environments, masks are key for preventing respiratory diseases, but their use has long faced a core challenge: maintaining facial microenvironmental thermal–humidity comfort while ensuring sufficient oxygen intake. The crux is respiratory forced convection, which disrupts the mask–face thermal–humidity balance, reduces heat exchange efficiency, and leaves traditional masks unable to reconcile ‘thermal–humidity regulation’ and ‘unobstructed breathing’.

Researchers have tried two approaches to address this problem; however, both suffer from major limitations: (i) using low thermal conductivity, low mid-infrared emissivity fabrics to enhance static insulation [2,3], but warp-weft woven fabrics’ open porous structure cannot suppress forced convective heat loss (0.5 m/s airflow causes >30% microenvironmental heat loss); and (ii) employing heat and moisture exchangers (HMEs) to adsorb exhaled waste heat and moisture via filters, yet these devices are large and heavy, have high respiratory resistance, and carbon dioxide-accumulating ‘dead spaces’, making them unsuitable for prolonged outdoor use [4].

**Figure 1.**
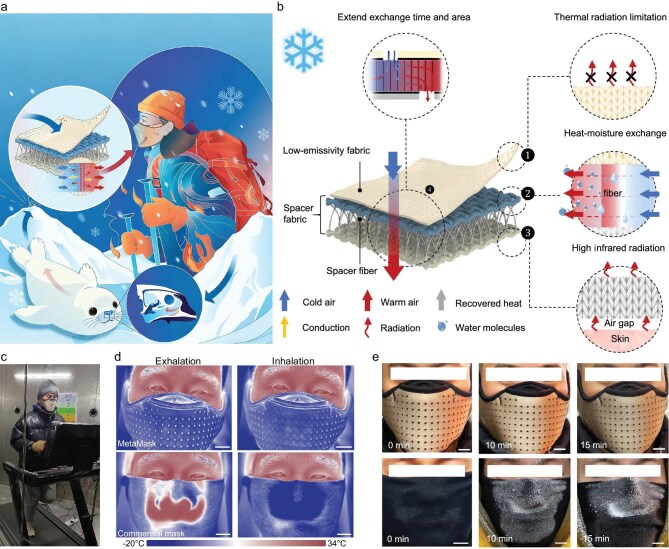
(a) Bioinspired design of MetaMask. (b) Heterogeneous structural design and thermal–humidity regulation mechanism of MetaMask. (c) Images of human locomotion in low-temperature environments. (d) Infrared images of subjects in inhalation and exhalation states while wearing the MetaMask and commercial masks (scale bar: 2 cm). (e) Frosting comparison between commercial masks and MetaMask surfaces at 0, 10, and 15 min (Scale bar: 2 cm). Reproduced with permission from Ref. [5]. Copyright 2025, Springer Nature.

In a recent study published in *Nature Communications*, Professor Tao’s group at Huazhong University of Science and Technology constructed a heterogeneous-structured mask (MetaMask) by drawing inspiration from the nasal turbinate structure of Arctic seals—an innovative practice of bionic design guided by the principles of functional materials engineering [5]. By integrating the strategies of ‘asymmetric radiation control’ and ‘condensation–evaporation synergy’, this design fundamentally breaks the inherent trade-off between thermal–humidity regulation and sufficient oxygen intake in traditional masks (Fig. 1a and b).

To systematically validate the practical application performance of MetaMask in cold environments, human subject experiments were conducted under low-temperature conditions (Fig. 1c). At −20°C, it elevates the temperature of inhaled air to 26.2°C, achieving a heat recovery rate of 82.1% ± 0.7% and a moisture recovery rate of 94.1% ± 1.1%—attesting to the efficacy of its structure-driven functional optimization (Fig. 1d). Leveraging efficient moisture recovery and a low water vapor transmission rate (0.13 g cm^−2^ day^−1^), MetaMask prevents significant surface frosting during continuous low-temperature use (Fig. 1e). Furthermore, the heated and humidified air delivered by MetaMask mitigates respiratory mucosal irritation, preventing post-exercise declines in forced expiratory volume in 1 s (FEV1) and cold-induced bronchoconstriction. Notably, its respiratory resistance is comparable to that of commercial masks, ensuring stable blood oxygen saturation (SpO_2_) during physical activity. Complemented by an ultra-high ultraviolet protection factor (UPF: 52 951), outstanding durability and self-cleaning properties, MetaMask is well suited for long-term deployment in complex scenarios such as high-altitude cold regions.

In summary, MetaMask resolves the inherent trade-off in cold-environment facial protection, addressing a pressing global public health challenge. Its rational structural design strategy exemplifies the transformative potential of integrating materials science with sports engineering—having provided technical support for China’s National Cross-country Skiing Team in the preparation for the 2022 Winter Olympics in Beijing. Furthermore, its scalable manufacturing process—requiring no custom equipment or high-cost processing—lowers the barrier for lab-to-industry translation, laying a foundation for future large-scale deployment. With its innovative design, robust performance and practicality, MetaMask advances the field of wearable protective equipment engineering and offers vital support to populations exposed to extreme cold, including athletes, military personnel, polar researchers and rescue workers. This work thus marks a significant step toward adapting to cold-related health threats, making a significant contribution to addressing public health security challenges in cold environments worldwide.
